# Nested-multiplex PCR detection of *Orthopoxvirus *and *Parapoxvirus *directly from exanthematic clinical samples

**DOI:** 10.1186/1743-422X-6-140

**Published:** 2009-09-11

**Authors:** Jônatas S Abrahão, Larissa S Lima, Felipe L Assis, Pedro A Alves, André T Silva-Fernandes, Marcela MG Cota, Vanessa M Ferreira, Rafael K Campos, Carlos Mazur, Zélia IP Lobato, Giliane S Trindade, Erna G Kroon

**Affiliations:** 1 Laboratório de Vírus, Departamento de Microbiologia, Instituto de Ciências Biológicas, Universidade Federal de Minas Gerais. Av. Antônio Carlos, 6627, caixa postal 486, CEP: 31270-901, Belo Horizonte, MG, Brazil; 2 Departamento de Medicina Veterinária Preventiva, Escola de Veterinária, Universidade Federal de Minas Gerais. Av. Antônio Carlos, 6627, CEP: 31270-901, Belo Horizonte, MG, Brazil; 3 Departamento de Microbiologia e Imunologia Veterinária, Universidade Federal Rural do Rio de Janeiro. BR465, Km07, Boa Esperança. CEP: 23890-000, Seropedica, Rio de Janeiro, Brazil

## Abstract

**Background:**

*Orthopoxvirus *(OPV) and *Parapoxvirus *(PPV) have been associated with worldwide exanthematic outbreaks. Some species of these genera are able to infect humans and domestic animals, causing serious economic losses and public health impact. Rapid, useful and highly specific methods are required to detect and epidemiologically monitor such poxviruses. In the present paper, we describe the development of a nested-multiplex PCR method for the simultaneous detection of OPV and PPV species directly from exanthematic lesions, with no previous viral isolation or DNA extraction.

**Methods and Results:**

The OPV/PPV nested-multiplex PCR was developed based on the evaluation and combination of published primer sets, and was applied to the detection of the target pathogens. The method showed high sensitivity, and the specificity was confirmed by amplicon sequencing. Exanthematic lesion samples collected during bovine vaccinia or contagious ecthyma outbreaks were submitted to OPV/PPV nested-multiplex PCR and confirmed its applicability.

**Conclusion:**

These results suggest that the presented multiplex PCR provides a highly robust and sensitive method to detect OPV and PPV directly from clinical samples. The method can be used for viral identification and monitoring, especially in areas where OPV and PPV co-circulate.

## Background

*Orthopoxvirus *(OPV) and *Parapoxvirus *(PPV) consist of large, enveloped, linear double-stranded DNA viruses, and are classified as genera of the family *Poxviridae *[1]. Several species included in these genera are related with worldwide acute exanthematic disease in humans and domestic animals, which cause serious economic losses and impact public health [1,2]. There are three zoonotic OPV species known, *Monkeypox virus *(MPXV), *Cowpox virus *(CPXV) and *Vaccinia virus *(VACV), and their presence is associated with an increased number of outbreaks in Africa, Europe, South America and Asia [3-6]. Similarly, several zoonotic PPV infections have been noted, and are caused mainly by *Bovine papular stomatitis virus *(BPSV), *Orf virus *(ORFV) and *Pseudocowpox virus *(PSCV) [7,8]. Even though humans are susceptible to MPXV, CPXV, VACV, BPSV, ORFV and PSCV, domestic animals such as sheep, goats, cats, dogs and dairy cattle can be infected by some OPV and/or PPV since the host-range of these viruses is large and incompletely known [9]. OPV and PPV transmission is usually promoted by fomites or direct contact, and the infected humans play an important role in viral spread among domestic animals, especially during milking and other occupational livestock activities [1,9].

Clinically, the exanthematic lesions caused by zoonotic OPV and PPV species are very similar, especially in humans and cows, and these can be critical for diagnosis in areas with OPV/PPV co-circulation [7,10-12]. Both OPV and PPV cause local or disseminated vesicular-pustular lesions that are associated with fever, lymphadenopathy, malaise and acute muscle pain [9]. Therefore, the OPV/PPV differential diagnosis involves serological, virological, microscopical and molecular techniques [5,7,8,11,13-16]. Although serological methods such as ELISAs, immunofluorescence assays and neutralization tests are useful and widely applied to OPV and PPV diagnosis, these techniques cannot differentiate anti-OPV antibodies resulting from acute infection from anti-OPV antibodies resulting from a prior vaccination [17]; additionally, the titer of anti-PPV neutralizing antibodies can promptly decrease to undetectable levels a few months after the infection [18]. Though the other molecular diagnostic approaches mentioned are also valuable and specific, they usually require viral isolation and/or DNA manipulation, and are designed to detect specifically OPV or PPV.

In the present work, we report the development of a nested-multiplex PCR system for the sensitive and reliable detection of OPV and PPV based on the combination and optimization of published primer sets. We also report its application for the detection of viruses included in these genera directly from bovine, ovine, caprine and human exanthematic lesions with no viral isolation or DNA manipulation. Sixty-eight clinical samples collected during Brazilian bovine Vaccinia (BV) or contagious ecthyma (CE) outbreaks were used to evaluate the performance of the OPV/PPV nested multiplex PCR and confirm its applicability to viral identification and monitoring.

## Methods and Results

### Multiplex PCR setting and sensitivity tests

The OPV/PPV multiplex PCR was designed based on computer simulation of different combinations of several published primer pairs, using software available online [19]. Two exclusive and highly conserved genes were targeted by nested-multiplex PCR: the OPV *viral growth factor *(*vgf*) and the PPV *major viral glycoprotein *(*b2l*); these genes have been widely used in OPV and PPV diagnosis and phylogenetic analysis (Table 1). The nested-multiplex PCR was carried out in a two-step reaction protocol. In the first step, the OPV primers vgfF and vgfR [20] were used in association with the PPV primers OVB2LF1 and OVB2LR1 [21]. In the nested step, a pair of internal OPV primers (vgfF2: ACACGGTGACTGTATCCA and vgfR2: CTAATACAAGCATAATAC) were designed from alignment of the vgf sequences of Brazilian VACV strains (Drumond and others, data not published) and other available OPV sequences (GenBank accession nos. [AY243312.1 (VACV-WR); AY678276.1 (VACV-LISTER); DQ792504.1 (Horsepox virus - HSPV); AY484669.1 (Rabbitpox virus - RPV); DQ437590.1 (VARV); AF482758.2 (CPXV)]); these primers were then used in association with the PPV primers PPP-1 and PPP-4 [7]. Several chemical and thermal conditions were evaluated. The best conditions were established based on amplicon yield and specificity [corresponding to the expected fragments of 170 bp (OPV) and 592 pb (PPV)], described as follows. In the first step, 2 μL of template were added to 18 μL of the PCR reaction mixture containing 0.4 mM of OPV primers (VGF-F and VGF-R), 0.8 mM of PPV primers (OVB2LF1 and OVB2LR1), 10 mM dNTPs, 2.0 mM MgCl_2_, 500 ng Bovine Serum Albumin (BSA) and 2 U of Taq DNA polymerase (Promega, Madison, USA), using the manufacturer's supplied 10× buffer. Reactions were performed with a DNA Mastercycler Epgradient (Eppendorf, Hamburg, Germany) using the following protocol: incubation at 95°C for 9 min; 30 cycles of denaturation (94°C, 1 min), annealing (45°C, 1 min) and extension (72°C, 1 min); final extension (72°C, 10 min). The nested PCR step was carried out using 1 μL of undiluted first PCR product as template. The same chemical and thermal conditions were used, but using internal OPV (vgfF2 and vgfR2 - 0.4 mM) and PPV (PPP-1 and PPP-4 - 0.8 mM) primers. The PCR products were electrophoresed on 8% PAGE gels and silver stained [22]. These same conditions were used in sensitivity tests. Some reactions were performed with the addition of both PPV and OPV scabs, with the purpose of simulating a possible co-infection. In order to confirm the OPV/PPV specificity, other exanthematic infectious agents were submitted to PCR: (i) a Bovine herpes virus positive scab kindly provided by Dr. Z. Lobato (Minas Gerais Federal University, Brazil), and (ii) a Brazilian *Sthaphylococcus aureus *strain, isolated from a hospital infection, kindly provided by Dr. L. Parucker (Santa Catarina Federal University, Brazil).

**Table 1:** Selected primers for the OPV/PPV nested-multiplex PCR

**Genus**	**Target gene**		**Primer sequence (5' - 3')**	**Reference**
**OPV**	*vgf*	*1st step*	vgfF: CGCTGCTATGATAATCAGATCATT	Fonseca et al., 1998
			vgfR: GATATGGTTGTGCCATAATTTTTAT	
				
		*Nested step*	vgfF2: ACACGGTGACTGTATCCA	This study
			vgfR2: CTAATACAAGCATAATAC	
				
**PPV**	*b2l*	*1st step*	OVB2LF1: TCCCTGAAGCCCTATTATTTTTGT	Hosamani et al., 2006
			OVB2LR1: GCTTGCGGGCGTTCGGACCTTC	
				
		*Nested step*	PPP-1: GTCGTCCACGATGAGCAG	Inoshima et al., 2000
			PPP-4: TACGTGGGAAGCGCCTCGCT	

The PCR sensitivity tests were performed using serial dilutions of the *vgf *and *b2l *external fragments cloned in the pGEM-T easy vector (Promega, Madison, WI, USA). These constructs were obtained by PCR amplification [20,21] from BV and CE outbreaks exanthematic lesions, followed by purification of the PCR products (QIAquick Gel Extraction Kit - QIAGEN, California - U.S.A.) and cloning into the pGEM-T easy vector. Three clones of each sample were sequenced in both orientations using M13 universal primers (Mega-BACE sequencer, GE Healthcare, Buckinghamshire, UK), and confirmed the PCR specificity. The *vgf *and *b2l *fragments were quantified (ND1000 spectrophotometer, Thermofisher Scientific - Massachusetts, U.S.A.) and submitted to OPV/PPV nested-multiplex PCR under distinct concentrations - 50, 25, 10, 5, 2 and 1 ng. The PCR products were electrophoresed on 8% PAGE gels and silver stained [22].

**Figure 1:**
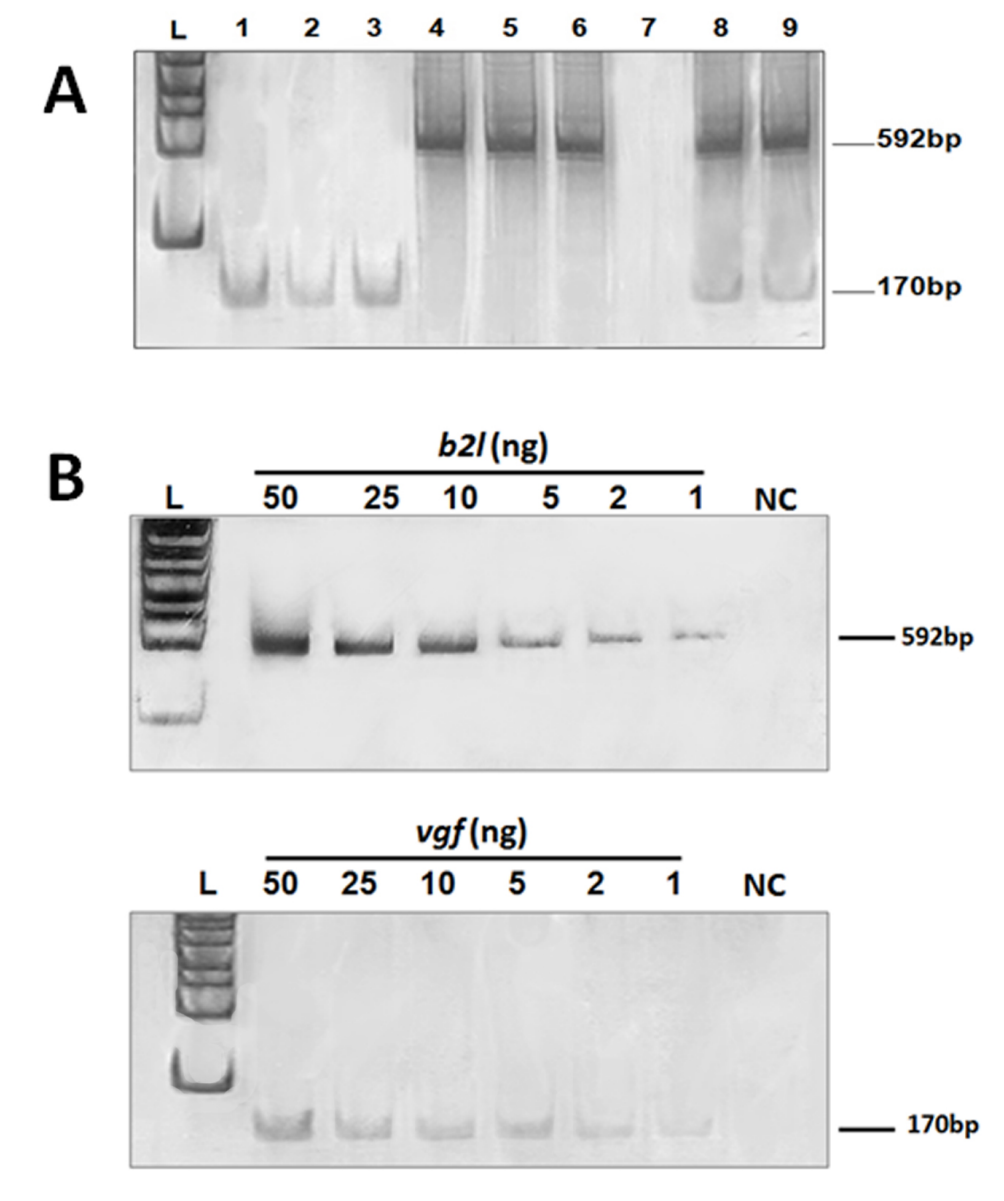
**(A) OPV/PPV nested-multiplex standardization and (B) sensitivity tests**. Exanthematic lesions from BV and CE outbreaks were used in PCR standardization and sensitivity assays. Different thermal and chemical conditions were tested. (A) lane 1-3: BV scabs and vesicles presenting OPV *vgf *gene amplification (170 bp); lane 4-6: CE scabs presenting PPV *b2l *gene amplification (592 bp); lane 7: negative control; lane 8-9: BV and CE scabs, simulating a possible co-infection, presenting the simultaneous amplification of OPV *vgf *and PPV *b2l *genes. (B) PCR sensitivity tests performed with different concentrations of *vgf *or *b2l *fragments. The nested-multiplex was able to detect OPV and PPV DNA until reactions in which there was 1 ng of *vgf *or *b2l *genes. The PCR products were electrophoresed on 8% PAGE gels and silver stained. NC: negative control.

Fragments of approximately 170 bp and 592 bp that correspond to the *vgf *and *b2l *genes, respectively, were specifically amplified best by PCR under the described thermal and chemical conditions (Figure 1-A). The amplified fragment sequences showed 100% identity with the VACV *vgf *gene (AY2433121 and others) or the ORFV *b2l *gene (FJ665818 and others). Reactions in which OPV and PPV scabs were added presented the amplification of both the170 and 592 bp fragments. No specific viral bands were observed in the negative control or in the in Bovine herpes virus and S. *aureus *reactions. Sensitivity tests using *vgf *or *b2l *cloned fragments presented unique and specific amplified bands of approximately 170 bp and 592 bp, respectively. In both cases, the PCR was able to detect until 1 ng of viral DNA fragment (Figure 1-B). No specific viral bands were observed in sensitivity test negative controls.

### Nested-multiplex applicability tests: clinical samples from exanthematic outbreaks

Vesicle contents and dried scabs from cattle udders and milkers' hands were collected during Brazilian BV outbreaks or from sheep and goats during CE outbreaks. This collection was accomplished using 1-ml insulin syringes, 0.45 mm×13 mm needles, and cotton swabs or a pair of forceps. Collected samples were chilled, transported to the laboratory, and stored at -70°C until processed. Vesicular liquid swabs were added to 200 μL of PBS and centrifuged at 2000 × g for 3 min. Scabs were macerated by a homogenizer (Politron, Littau, Switzerland) in PBS (0.1 g scab/0.9 mL PBS) and clarified by centrifugation at 2000 × g for 3 min. Two microliters of the supernatants were used in the nested-multiplex PCR. Some expected PCR products were directly sequenced (ET Dynamic Terminator for MegaBACE - GE Healthcare, Fairfield, USA) and compared with available GenBank sequences using an online blast program . To avoid any possibility of laboratory cross-contamination, the different samples were manipulated separately.

A total of 64 clinical samples were collected and then submitted to OPV/PPV nested-multiplex PCR (Table 2). Of these samples, 56 were collected during BV outbreaks (36 from bovines and 20 from humans) and 8 samples were collected during CE outbreaks (3 from caprines and 5 from ovines). All collected BV and CE clinical samples were previously tested by other molecular methods (Fonseca et al., 1998; Inoshima et al., 2000) and were confirmed VACV and ORFV infections, respectively. Among the BV clinical samples, the OPV/PPV nested-multiplex PCR detected OPV DNA in 53 scabs/vesicles (94.4%). The multiplex was able to detect PPV DNA in all analyzed CE clinical samples. Considering all bovine, human, ovine and caprine samples, the nested-multiplex PCR presented a positivity of 95.3%. The sequences of the amplified fragments again confirmed the PCR specificity, showing high identity with the VACV *vgf *gene or the ORFV *b2l *gene sequences. No co-infection case was detected in this molecular screening.

**Table 2:** Clinical samples used to evaluated the performance of the OPV/PPV nested-multiplex PCR

**State/Year**		**N° of specimens**	**Source^a^**	**Designation**	**Specimen**	**Positive samples**	**Result**	**Reference**
Minas Gerais, 2005		2	B	GP1V, GP2V	scab	2	OPV	Trindade et al., 2006
Minas Gerais, 2005		11	B/H	SV	scab and vesicle	10	OPV	Trindade et al., 2007
Minas Gerais, 2003		1	B	PSTV	scab	1	OPV	Leite et al., 2005
Minas Gerais, 2005		13	B/H	MARV	scab and vesicle	11	OPV	Abrahão et al., upubl. Data
Espírito Santos, 2008		4	B/H	LINV	scab and vesicle	5	OPV	Abrahão et al., upubl. Data
Minas Gerais, 2005		5	B/H	RPLV	scab and vesicle	5	OPV	Abrahão et al., upubl. Data
Minas Gerais, 2005		8	B/H	JQRV	scab and vesicle	7	OPV	Abrahão et al., upubl. Data
Minas Gerais, 2008		8	B	PRGV	scab	8	OPV	Abrahão et al., upubl. Data
Minas Gerais, 2008		4	H	ARGV	vesicle	4	OPV	Abrahão et al., upubl. Data
Minas Gerais, 1990		1	C	ORF-A	sacb	1	PPV	Mazur & Machado, 1990
Pernambuco, 1993		2	C	NE1, NE2	scab	2	PPV	Mazur et al., 2000
Mato Grosso, 2005		5	O	MT05	scab	5	PPV	Abrahão et al., 2009
	**Total**	**64**	**Positivity 61 (95,31%)**					

^**a **^B = bovine; H = human; C = caprine; O = ovine

## Conclusion

In the present work, the creation of a multiplex PCR method for the simultaneous detection of OPV and PPV has been described and tested with exanthematic clinical samples from distinct viral hosts, with no DNA extraction or virus manipulation. The method proposed was able to correctly identify the target pathogens by amplification of conserved genes, even in co-infection simulations. The primer selection and multiplex optimization allowed the creation of a robust method, with performances comparable to conventional one-pathogen PCR assays [7,20]. The sequencing of *vgf *and *b2l *amplicons confirmed the specificity of the nested-multiplex approach. The sensitivity and robustness of the proposed method, together with its ability to perform well on exanthematic clinical samples, make it a suitable method to rapidly identify and effectively monitor OPV and PPV infection outbreaks.

## Competing interests

The authors declare that they have no competing interests.

## Authors' contributions

JSA, LSL, GST and EGK participated in the planning of the project. EGK was the leader of the project. ZIPL and CM collected the samples. JSA, LSL, FSS, PAA, ATSF, MMGC, VMF and RCK performed the PCR and phylogenetic analysis. All authors read and approved the final manuscript.
